# Rhizosphere Microbiome Dynamics Associated with Root Rot in *Polygonatum kingianum* Coll.

**DOI:** 10.3390/microorganisms14071568

**Published:** 2026-07-17

**Authors:** Shanshan Xu, Shuangfei Deng, Jielin Shi, Meiqi Huang, Rui Shi

**Affiliations:** Yunnan Provincial Key Laboratory for Conservation and Utilization of In-Forest Resource, College of Forestry, Southwest Forestry University, Kunming 650224, China; xuss2024@swfu.edu.cn (S.X.);

**Keywords:** *Polygonatum kingianum*, root rot, rhizosphere microbiome, FUNGuild, co-occurrence network, soil physicochemical properties, Yunnan Province

## Abstract

*Polygonatum kingianum* Coll. (PKC) is a valuable medicinal herb native to Yunnan, China, but its yield and quality are severely threatened by root rot. To elucidate the rhizosphere microbial dynamics associated with this disease and to identify location-transcending patterns, we collected rhizosphere soils from healthy and diseased PKC plants at three planting bases in Lincang, Qujing, and Kunming. Soil properties were measured, and bacterial (16S rRNA) and fungal (ITS) communities were characterized by amplicon sequencing. Geographic origin emerged as the dominant factor associated with microbial community structure, with soil moisture, organic matter, and nutrients all showing significant associations. The effect of disease on microbial diversity was site-specific. Nevertheless, LEfSe analysis identified cross-location-consistent indicator genera: *Acidiferrimicrobium* and HSB_OF53_F07 were enriched in healthy soils, while *Burkholderia-Caballeronia-Paraburkholderia* and *Rhodanobacter* were enriched in diseased samples. At the trophic mode level, a consistent pattern was observed: diseased rhizosphere soils had higher relative abundance of pathotrophic fungi and lower abundance of symbiotrophic fungi compared to healthy soils. However, at the finer guild level, no individual guild showed statistically significant differences after FDR correction. Co-occurrence network analysis revealed a striking structural reorganization: healthy plants harbored a fungus-dominated network (57.04% fungi), whereas diseased plants shifted to a bacterium-dominated network (55.71% bacteria), accompanied by an increased proportion of negative correlations. Redundancy analysis (RDA) and Mantel tests further confirmed that soil physicochemical properties, rather than health status, were the primary factors associated with microbial community variation (Mantel’s r = 0.725 for bacteria, 0.768 for fungi). Collectively, this study provides the first systematic evidence of cross-location common microbial shifts and network reorganization associated with PKC root rot. These findings offer a microecological basis for developing green prevention and control strategies against this devastating disease.

## 1. Introduction

*Polygonatum kingianum* Coll. (PKC) is a perennial herb of the genus Polygonatum (family Liliaceae), primarily distributed in the southwestern region centered on Yunnan Province. It is an important authentic medicinal material in Yunnan [1]. This plant is rich in various active ingredients beneficial to the human body. In traditional Chinese medicine, PKC is recognized for its properties of nourishing the kidney and essence, moistening yin and reducing dryness; modern pharmacological research further confirms that PKC has multiple medicinal values, such as anti-aging, immune enhancement, hematopoiesis regulation, anti-inflammatory, anti-tumor, and hypoglycemic effects [2].

However, with the continuous expansion of cultivation areas and long-term continuous cropping, the problem of soil microecological imbalance has become increasingly severe, and frequent diseases have become a key bottleneck restricting its yield and quality. Common diseases of PKC include black spot, gray mold, root rot, and leaf spot [3]. Among them, root rot is a devastating soil-borne disease. Its causative fungi can spread rapidly through soil and water movement. Once infected plants appear in the field, they are prone to causing large-scale infection, resulting in severe yield reduction or even total crop failure [4], posing a significant threat to the sustainable development of the PKC industry. Root rot of PKC is caused by multiple fungal pathogens, including *Fusarium oxysporum*, *Fusarium redolens*, *Fusarium concentricum*, and *Aspergillus awamori*, with *Fusarium* species being the most frequently isolated and considered the dominant pathogens [3,4]. However, root rot is rarely the result of a single pathogen; rather, it involves a complex pathobiome—the assemblage of pathogenic microorganisms and their interacting microbial associates that collectively contribute to disease development [5]. Moreover, disease occurrence is often preceded or accompanied by rhizospheric dysbiosis, defined as a disruption of the native rhizosphere microbial community structure and function that weakens the soil’s natural disease-suppressive capacity and facilitates pathogen invasion [6]. These integrative concepts emphasize that root rot should be understood not merely as pathogen infection but as a microecological imbalance involving multi-kingdom interactions and environmental triggers.

Rhizosphere microorganisms are key drivers of soil energy flow, organic matter turnover, and nutrient cycling and are the most active components in rhizosphere soil ecosystems [7]. The changes in community diversity and functional diversity not only affect the dynamic changes in soil quality and ecological environment conditions [8], but are also closely related to the occurrence of soilborne diseases. On the one hand, in soils with rich microbial diversity, pathogenic microorganisms find it more difficult to survive; on the other hand, the occurrence of soilborne diseases can further reduce the diversity of soil microorganisms [9]. Meanwhile, the structure of soil microbial communities can effectively reflect the nutrient status of soil and the health and stability level of ecosystems [10]. Research has shown that pathogenic microorganisms in rhizosphere soil are the main source of soilborne diseases [11]. They induce diseases by infecting host plants and altering interspecific competition patterns [12]. The diversity and relative abundance of rhizosphere microorganisms are closely related to the occurrence of plant diseases. Numerous studies have shown that a rich and stable rhizosphere microbial community can effectively suppress soilborne diseases [13,14]. In addition, the composition of rhizosphere microbial communities will dynamically adjust with changes in plant species, developmental stages, and environmental conditions. Generally speaking, the more complex the microbial community structure, the more stable the physical and chemical properties of soil tend to be [15]; conversely, the imbalance of rhizosphere microorganisms directly associated with plant health and soil fertility levels [16]. It is worth noting that non-biological factors in soil, such as organic matter content, pH value, humidity, etc., also significantly correlate with the activity and spread of pathogenic microorganisms, thereby being associated with the frequency and severity of soilborne diseases [17]. In summary, the systematic analysis of the impact mechanisms of soil biotic and abiotic factors on disease occurrence is the basis for formulating scientific and effective disease prevention and control strategies.

Although several recent studies have begun to characterize the rhizosphere microbiome of Polygonatum species, most have focused on *P. cyrtonema* or *P. sibiricum*. For instance, Pang et al. (2022) showed that root rot disrupted the microbial communities and reduced diversity in the *P. cyrtonema* rhizosphere [18]. Among the limited studies on *P. kingianum*, one investigation across five planting areas focused on the relationship between microbial communities and herb quality, identifying soil properties as key predictors of microbial structure [19]. However, research specifically addressing the rhizosphere microbial dynamics associated with root rot in PKC remains scarce, especially whether there are common microbial change patterns in different regions and how soil physicochemical factors are associated with these changes, which lacks a systematic understanding. To address this gap, we formulated the following hypotheses: (i) PKC root rot is associated with consistent, location-transcending shifts in rhizosphere microbial community composition and function; (ii) specific bacterial and fungal taxa serve as cross-location indicator genera for healthy versus diseased status; and (iii) the disease is accompanied by fundamental reorganization of microbial interaction networks and trophic functional profiles. To this end, this study selected three Yunnan PKC planting bases with different geographical locations, collected healthy and infected root rot plants’ rhizosphere soil, and analyzed the bacterial and fungal community composition in the rhizosphere soil using amplicon sequencing technology. Combined with quantitative determination of soil physicochemical properties, the aim was to identify the microbial community changes that are significantly related to root rot occurrence across the three locations and reveal the key soil physicochemical factors associated with these microbial changes. The research results will provide a microecological basis for further revealing the pathogenesis of PKC root rot and provide theoretical guidance and practical reference for the green prevention and control of this disease.

## 2. Materials and Methods

### 2.1. Sample Collection

Three PKC planting bases were selected as sampling points in Yunnan Province, namely: Hejiazhuang planting base in Fengqing County, Lincang City (99.31° E, 24.13° N), Zhehai Linxia industrial base in Huize County, Qujing City (25.75° N, 103.55° E), and Dayang Fengle Linxia planting base in Xundian County, Kunming City (103.20° E, 25.46° N). At each base, healthy and diseased plants coexisted in the same field, and rhizosphere soil samples were collected from both groups. Plants were diagnosed as diseased based on typical symptoms: wilting, leaf yellowing, rhizome browning and necrosis, water-soaked lesions, and soft rot with foul odor; healthy plants showed no such symptoms (see Supplementary Figure S1 for contrasting images). Rhizosphere soil was collected by brushing the soil tightly adhering to the root surface (1–2 mm thickness) after removing loose soil. At each site, five healthy and five diseased plants were sampled. For each group, the rhizosphere soils from all five plants were pooled, thoroughly homogenized, and divided into three aliquots as biological replicates. After passing through a 0.2 mm sieve, the collected soil samples were immediately divided and frozen in liquid nitrogen before being transferred to a −80 °C freezer for storage. Bulk soil samples (without plants) were not collected. The grouping markers for each sample are as follows: Kunming healthy plant rhizosphere soil (DZJK), Kunming diseased plant rhizosphere soil (DZGF), Lincang healthy plant rhizosphere soil (FSJK), Lincang diseased plant rhizosphere soil (FSGF), Qujing healthy plant rhizosphere soil (AHJK), Qujing diseased plant rhizosphere soil (AHGF).

### 2.2. Soil Physicochemical Property Testing

Soil water content (WC) was determined gravimetrically by drying soil at 105 °C to constant weight. The soil pH value was measured according to the method described by Ji et al. [20]. Total nitrogen content (TN) was analyzed using a dry combustion method with an elemental analyzer (Vario MACRO cube, Elementar, Germany). Total phosphorus (TP) and total potassium (TK) were measured using colorimetric and flame atomic absorption spectrophotometry, respectively [21]. According to Shigaki and Sharpley’s [22] study, available phosphorus (AP) was extracted using sodium bicarbonate, followed by ammonium acetate (NH4AC) extraction. Alkaline nitrogen (AN) was determined using the alkaline diffusion method [23], and available potassium concentration (AK) was measured using a flame photometer [24]. A soil nutrient rapid analyzer (ST-TR02, Qingdao, China) was used to measure soil organic matter (SOM) [25]. All soil physicochemical data were statistically analyzed using one-way ANOVA with Tukey’s HSD post hoc test to compare all six groups, and independent sample *t*-tests were used for paired comparisons between healthy and diseased samples within each location. Significance was set at *p* < 0.05.

### 2.3. DNA Extraction, Amplification, and Sequencing

Total genomic DNA was extracted from 0.5 g of soil using the TIANamp Soil DNA Kit (Tiangen, Beijing, China) according to the manufacturer’s instructions. After passing the purity test using a NanoDrop-2000 spectrophotometer (Thermo Fisher Scientific, Wilmington, DE, USA), the DNA was stored at −20 °C for future use. Amplification of bacterial 16S rRNA gene V3–V4 region and fungal ITS rRNA gene ITS1-1F region using primer pairs: bacterial 341F (5′-CCTAYGGGRBGCASCAG-3′) and 806R (5′-GACTACNNGGGTATCTAAT-3′); fungal ITS1-1F-F (5′-CTGGTCATTTAGGAGAGATA-3′) and ITS1-1F-R (5′-GCTGTGTTCATGATGC-3′) [26]. The amplicon library was prepared using the Nextera XT Index Kit (Illumina Inc., Madison, WI, USA) according to the 16S and ITS metagenomic sequencing library construction protocol. The library quality was evaluated through gel electrophoresis, 1 × AMPure XP magnetic bead purification, and Agilent Bioanalyzer 2100 system (Agilent Technologies, Santa Clara, CA, USA) (with DNA 1000 chip). The purified product was quantified using a Qubit 2.0 fluorescence meter and a dsDNA detection kit (Life Technologies, Carlsbad, CA, USA; item number: Q328520) [27]. After mixing equal amounts of amplicon libraries, sequencing was completed by Beijing Novogene Bioinformatics Technology Co., Ltd. (Beijing, China). on the Illumina MiSeq platform. A total of 36 samples (6 groups × 3 replicates × 2 amplicon types) were sequenced.

### 2.4. Data Quality Control, OTU Clustering, and Species Annotation

The raw sequencing data was output in FASTQ format. Trimmomatic software (v0.32) was used to remove low-quality read segments (quality value < 20) and organize them into a double-ended form [28]. The dual-end read segments were concatenated using FLASH software (v1.2.11), with a minimum overlap of 10 bp, a maximum overlap of 200 bp, and a maximum mismatch rate of 20% [29]. After trimming and joining, the final read length was approximately 420–460 bp for bacterial 16S and 350–400 bp for fungal ITS. Chimeric sequences were removed using UCHIME software (v4.2) to obtain clean reads for subsequent analysis [30]. Based on clean read segments, the UPARSE process was used to cluster operational taxonomic units (OTUs) with a similarity threshold of 97% [31]. The classification annotation of bacterial sequences relied on the SILVA database (SILVA v138.1, confidence threshold of 70%), while fungal sequences relied on the UNITE database (v8.3), and taxonomic assignment was performed using the Ribosome Database Project (RDP) classifier [32,33].

### 2.5. Statistical Analysis and Visualization

The QIIME software (version 1.9.1) was used to calculate the diversity index, including the number of observed OTUs, Chao1 richness estimates, Shannon diversity index, and abundance-based coverage estimates (ACE) [34]. The beta diversity assessment first calculated the Bray–Curtis dissimilarity index for bacteria and weighted UniFrac distance for fungi, and then performed principal coordinate analysis (PCoA) in QIIME based on these indices. Use relevant scripts in R software (version 4.2.0) to draw bar charts and heatmaps of relative abundance at the genus level [35]. All statistical analyses were performed using R version 4.2.0. Alpha diversity indices (Chao1 and Shannon) were compared between groups using the Wilcoxon rank-sum test. Beta diversity differences were assessed using PERMANOVA (adonis function in vegan package) with 999 permutations. To identify differentially abundant taxa between healthy and diseased groups, LEfSe (Linear Discriminant Analysis Effect Size) analysis was performed using the online Galaxy platform (https://bitbucket.org/biobakery/biobakery/wiki/lefse, accessed on 2 June 2026). Briefly, the non-parametric Kruskal–Wallis rank-sum test was first applied to detect taxa with significant abundance differences among groups (*p* < 0.05). Next, Wilcoxon rank-sum test was used for pairwise comparisons between groups. Finally, linear discriminant analysis (LDA) was performed to estimate the effect size of each differentially abundant taxon, with a logarithmic LDA score threshold > 2.0 considered statistically significant [36].

Based on OTU-level data, the SPARCC method in R was used for co-occurrence network analysis. Only taxa with relative abundance > 0.01% in at least three samples were retained. Strong and significant correlations were defined as Spearman’s correlation coefficient |ρ| > 0.6 with *p* < 0.01 (after FDR correction). The calculation and visualization of network features were completed using Gephi software (version 0.9.2) [37]. An independent sample *t*-test was used for inter-group comparison, with a significance level set at *p* < 0.05. All statistical calculations were performed in IBM SPSS software (version 20.0). The graphic post-processing and illustration production were unified using Adobe Illustrator CC 2019 (Adobe Systems Inc., San Francisco, CA, USA).

Mantel tests based on Spearman’s rank correlation with 999 permutations were performed using an online platform (https://www.cloudtutu.com, accessed on 25 May 2026) to evaluate the overall relationship between soil physicochemical property distance matrix (Euclidean distance of nine normalized variables: WC, SOM, TN, TP, TK, AN, AP, AK, pH) and microbial community distance matrix (Bray–Curtis distance for bacteria, weighted UniFrac distance for fungi) [38]. Spearman’s rank correlation analysis was performed to evaluate the relationships between individual soil physicochemical properties and the relative abundance of microbial genera (mean relative abundance > 0.1%). *p*-values were adjusted for multiple comparisons using the false discovery rate (FDR) method (Benjamini–Hochberg) [39]. Correlations with |ρ| > 0.6 and FDR-adjusted *p* < 0.05 were considered statistically significant. The complete correlation matrices are provided in Supplementary Tables S1 and S2. For comparisons of relative abundance at phylum and genus levels between healthy and diseased groups, Wilcoxon rank-sum tests were performed with FDR correction (Benjamini–Hochberg, q < 0.05). Only taxa with q < 0.05 were considered significantly different between groups. To further assess the overall relationships between soil properties and microbial communities, Mantel tests based on Spearman’s rank correlation (999 permutations) were performed between: (i) the soil physicochemical property distance matrix and the microbial community composition distance matrix (Bray–Curtis for bacteria, weighted UniFrac for fungi); (ii) the soil property distance matrix and the genus-level abundance distance matrix (Bray–Curtis) for both bacteria and fungi; and (iii) the soil property distance matrix and alpha diversity indices (Chao1 and Shannon) for both bacteria and fungi.

For bacterial functional prediction, PICRUSt2 was used to predict KEGG pathway abundances (level 3) based on the 16S rRNA gene sequences [40]. Differentially abundant pathways between healthy and diseased groups at each location were identified using Welch’s *t*-test with false discovery rate (FDR) correction (Benjamini–Hochberg, q < 0.05). For fungal functional prediction, FUNGuild was used to assign trophic modes and guilds to fungal OTUs [41]. Differences in trophic mode and guild abundances between groups were tested using the Wilcoxon rank-sum test with FDR correction (q < 0.05).

## 3. Results

### 3.1. Soil Physicochemical Properties Associated with Healthy and Diseased PKC Rhizosphere

According to the analysis of the physicochemical properties of healthy and diseased plant rhizosphere soil in Kunming (DZ), Lincang (FS), and Qujing (AH), geographical origin is the dominant factor associated with soil properties, and the three regions show significant differences in most indicators (Table 1). Statistical comparisons (one-way ANOVA with Tukey’s HSD and independent sample *t*-tests) confirmed these differences, with superscript letters in Table 1 indicating significant differences among groups (*p* < 0.05). For example, the organic matter, total nitrogen, and available phosphorus content of Kunming soil are significantly higher than those of the other two regions, while the total potassium and available potassium content of Lincang soil are the highest. The contrasting morphology between healthy and diseased plants is illustrated in Supplementary Figure S1.

The association between health and disease status on physicochemical properties was site-specific. According to paired *t*-tests (*p* < 0.05), in Lincang, the moisture content of diseased soil was significantly higher than that of healthy soil, while organic matter, total nitrogen, and available nitrogen were significantly lower. In Kunming, diseased soil also showed a significant increase in moisture content by paired *t*-test (*p* < 0.05), but no significant differences were observed in other nutrient indicators. In Qujing, there were no significant differences in any of the nine physicochemical indicators between healthy and diseased soils (paired *t*-tests, all *p* > 0.05). The pH values of all samples were neutral to slightly alkaline (7.16–7.30) with no inter-group differences. It should be noted that the conservative Tukey’s HSD test (Table 1), which adjusts for multiple comparisons across all six groups, may not always detect these site-specific paired differences; therefore, the letters in Table 1 primarily reflect geographic variation, while the within-site comparisons are supported by independent-sample *t*-tests as described above. These results indicate that the association between root rot on soil physicochemical properties is highly site-specific and does not follow a universal pattern.

### 3.2. Microbial Alpha and Beta Diversity

**Figure 1:**
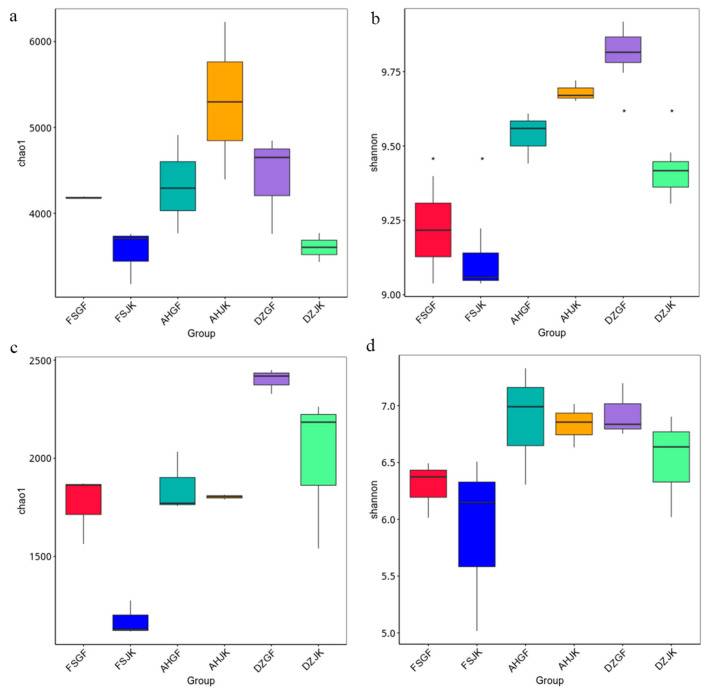
Alpha diversity indices of bacterial (**top**) and fungal (**bottom**) communities in healthy (JK) and root rot-affected (GF) rhizosphere soils of PKC collected from three geographic locations (FS, AH, DZ). (**a**) Bacterial Chao1 richness, (**b**) Bacterial Shannon diversity, (**c**) Fungal Chao1 richness, (**d**) Fungal Shannon diversity. Values are shown as mean ± SD (*n* = 3). Asterisks indicate significant differences between healthy and diseased groups within each location (* *p* < 0.05, *p* < 0.01, Wilcoxon rank-sum test). Abbreviations: FS, Lincang (Fengqing County); AH, Qujing (Huize County); DZ, Kunming (Xundian County); JK, healthy plants; GF, root rot-affected (diseased) plants.

To evaluate the species richness and diversity of bacterial and fungal communities across different groups, we calculated the Chao1 and Shannon indices (Figure 1). For bacterial communities, the Chao1 index (Figure 1a) indicated that AHJK (5704.83 ± 325.61) and DZGF (4619.47 ± 412.85) exhibited the highest species richness, while FSJK (3366.72 ± 298.54) and DZJK (3279.15 ± 361.42) had the lowest. The Shannon index (Figure 1b) showed that DZGF (3.89 ± 0.21) and DZJK (3.85 ± 0.19) had the highest diversity, while FSJK had the lowest (2.05 ± 0.17). Wilcoxon rank-sum tests revealed that bacterial Shannon diversity was significantly lower in diseased samples than in healthy samples at Lincang (FS) and Kunming (DZ) (*p* < 0.05), but not at Qujing (AH) (*p* > 0.05) [42]. For fungal communities, the Chao1 index (Figure 1c) showed that DZGF (2412.36 ± 356.72) and DZJK (2215.48 ± 298.53) had the highest species richness, while FSGF (1763.25 ± 312.47) and FSJK (1628.91 ± 287.36) had the lowest. The Shannon index (Figure 1d) showed that AHGF (7.16 ± 0.23) and DZGF (7.04 ± 0.19) had the highest fungal diversity, while FSJK (6.53 ± 0.27) and DZJK (6.67 ± 0.24) had relatively lower levels.

**Figure 2:**
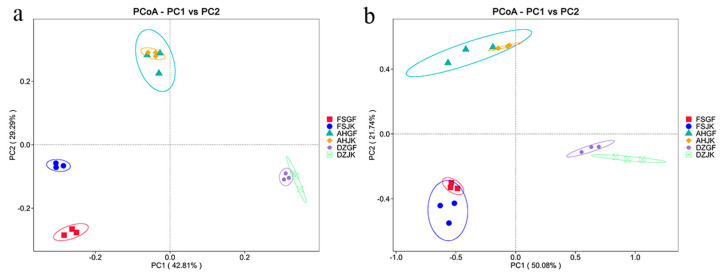
Beta diversity analysis. (**a**) PCoA of bacterial communities based on Bray–Curtis distance; (**b**) PCoA of fungal communities based on weighted UniFrac distance. PERMANOVA results (999 permutations): for bacteria, geographic location R^2^ = 0.682, *p* = 0.001; health status R^2^ = 0.073, *p* = 0.089 (not significant). For fungi, geographic location R^2^ = 0.651, *p* = 0.001; health status R^2^ = 0.068, *p* = 0.102 (not significant). Abbreviations: FS, Lincang (Fengqing County); AH, Qujing (Huize County); DZ, Kunming (Xundian County); JK, healthy plants; GF, root rot-affected (diseased) plants.

To evaluate beta diversity, we performed principal coordinate analysis (PCoA) on bacterial (16S) and fungal (ITS) communities (Figure 2) [43]. For bacterial communities, PCoA based on Bray–Curtis distance (Figure 2a) showed that PCo1 and PCo2 explained 42.81% and 29.29% of the total variation, respectively. Samples showed clear geographical clustering: Kunming (DZ) samples clustered in the positive direction of PCo1, Lincang (FS) samples in the negative direction of PCo1, and Qujing (AH) samples in the negative direction of PCo2. Notably, healthy and diseased samples from the same location did not form independent clusters. For fungal communities, PCoA based on weighted UniFrac distance (Figure 2b) showed that PC1 and PC2 explained 50.08% and 21.74% of the total variation. Similarly to bacteria, fungal samples were also significantly separated by geographical origin. These results indicate that geographic origin is the dominant factor associated with rhizosphere microbial community structure, as further confirmed by PERMANOVA (for bacteria: R^2^ = 0.682 for location vs. R^2^ = 0.073 for health status, *p* = 0.001; for fungi: R^2^ = 0.651 for location vs. R^2^ = 0.068 for health status, *p* = 0.001), while plant health status has a comparatively weaker and site-specific effect.

### 3.3. Microbial Community Composition at Phylum and Genus Levels

**Figure 3:**
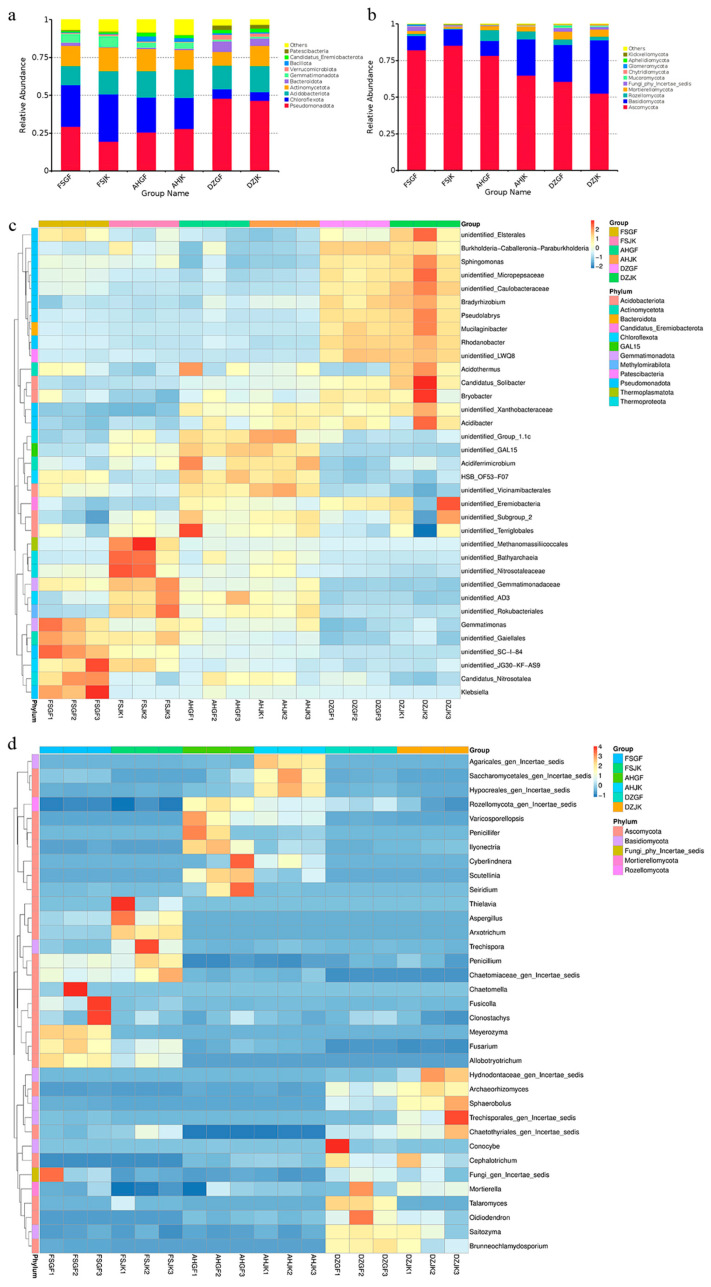
Composition of rhizosphere microbial communities in PKC at phylum and genus levels. (**a**,**b**) Relative abundance of bacterial (16S) and fungal (ITS) communities at the phylum level, with paired bars for healthy (JK) and diseased (GF) samples at each location to facilitate direct visual contrast. (**c**,**d**) Heatmaps of bacterial and fungal communities at the genus level; color intensity represents Z-score-standardized relative abundance (blue: lower, red: higher). For both phylum- and genus-level data, samples from the same geographic location (FS, AH, or DZ) cluster together regardless of health status, while samples from different locations show clear separation, indicating that geographic origin is more strongly associated with microbial community structure than disease status. Each group contains 3 biological replicates. Differences in relative abundance between groups were assessed by Wilcoxon rank-sum tests with FDR correction (q < 0.05); taxa with q < 0.05 are discussed as significantly different in the text. Abbreviations: FS, Lincang (Fengqing County); AH, Qujing (Huize County); DZ, Kunming (Xundian County); JK, healthy plants; GF, root rot-affected (diseased) plants.

At the phylum level, bacterial and fungal community compositions showed significant differences among samples from different geographical locations (Figure 3a,b). For bacterial communities, Proteobacteria and Acidobacteria were the dominant phyla across all three sites. However, the relative abundance of Proteobacteria was higher in Kunming (DZ) samples compared to Lincang (FS) and Qujing (AH) samples, while Acidobacteria was more abundant in Lincang samples (Wilcoxon rank-sum test, FDR-adjusted q < 0.05). Notably, approximately 75% of bacterial sequences could not be classified at the phylum level, suggesting the presence of numerous uncultured or novel bacterial taxa. For fungal communities (Figure 3b), Ascomycota occupied an absolutely dominant position in all samples, with relative abundance exceeding 70%, while Basidiomycota and Mortierellomycota were present at lower abundances.

At the genus level, we constructed heatmaps to visualize the relative abundance patterns (Figure 3c,d). The bacterial heatmap revealed that *Burkholderia-Caballeronia-Paraburkholderia*, *Rhodanobacter*, and *Pseudolabrys* had higher relative abundances in healthy groups (FSJK, AHJK, DZJK) compared to their corresponding diseased groups (FSGF, AHGF, DZGF) (Wilcoxon rank-sum test, FDR-adjusted q < 0.05). Conversely, genera such as *Acidiferrimicrobium*, HSB_OF53_F07, and *Bryobacter* were more abundant in diseased rhizosphere soil (q < 0.05). The fungal heatmap showed that *Fusarium* was highly abundant across all samples, with no significant differences between healthy and diseased plants (q > 0.05 for all pairwise comparisons), while genera such as *Meyerozyma*, *Allobotryotrichum*, and *Fusicolla* exhibited higher relative abundance in diseased plants at the Lincang and Kunming sites (q < 0.05).

### 3.4. Differential Microbial Genera Associated with Root Rot

**Figure 4:**
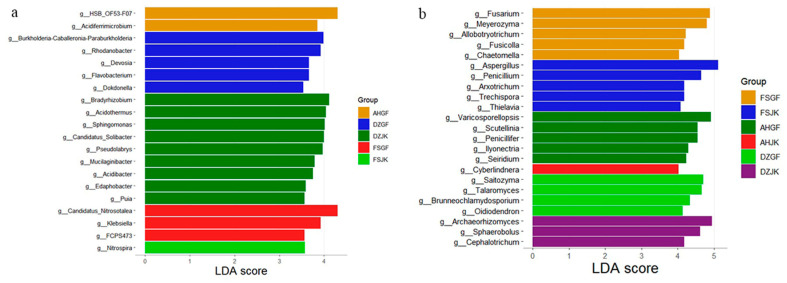
Linear discriminant analysis (LEfSe) of bacterial (**a**) and fungal (**b**) communities in rhizosphere soils of healthy and root rot-affected PKC. Only taxa with significant differential abundance (Kruskal–Wallis test, *p* < 0.05) and LDA scores > 2.0 are shown. Colors indicate different groups (see legend), and bar length represents the LDA score. Abbreviations: FS, Lincang (Fengqing County); AH, Qujing (Huize County); DZ, Kunming (Xundian County); JK, healthy plants; GF, root rot-affected (diseased) plants.

To identify differential indicator microorganisms across different groups, we conducted LEfSe analysis as described in Section 2.5 with a logarithmic LDA threshold > 2.0 [44]. A total of 19 bacterial indicator genera with significant differences in abundance among the six groups were identified (Figure 4a). In Lincang, *Acidiferrimicrobium* and HSB_OF53_F07 were significantly enriched in the healthy group (FSJK), whereas *Burkholderia-Caballeronia-Paraburkholderia* and *Rhodanobacter* showed higher LDA scores in the diseased group (FSGF). Similar patterns were observed in Qujing and Kunming, with slight variations in LDA scores. In Qujing, the healthy group (AHJK) showed high LDA scores for both *Acidiferrimicrobium* and HSB_OF53_F07, while the diseased group (AHGF) was enriched with *Burkholderia-Caballeronia-Paraburkholderia* and *Pseudolabrys*. In Kunming, the diseased group (DZGF) showed significant enrichment of *Burkholderia-Caballeronia-Paraburkholderia* and *Rhodanobacter*, while the healthy group (DZJK) was primarily indicated by *Acidiferrimicrobium* and HSB_OF53_F07 (LDA = 4.1). These cross-location consistent enrichment patterns suggest that these genera may serve as potential biological indicators for rhizosphere health in PKC. For the fungal community (Figure 4b), a total of 23 significantly different indicator genera were identified. However, in contrast to bacteria, *Fusarium* showed extremely high LDA scores across all six groups without significant inter-group specificity. Similarly, genera such as *Meyerozyma*, *Allobotryotrichum*, and *Fusicolla* were highly enriched in all groups. These results suggest that these fungal genera are likely resident core microbiota in the PKC rhizosphere rather than disease-specific indicators.

### 3.5. Cross-Kingdom Co-Occurrence Network Analysis

**Figure 5:**
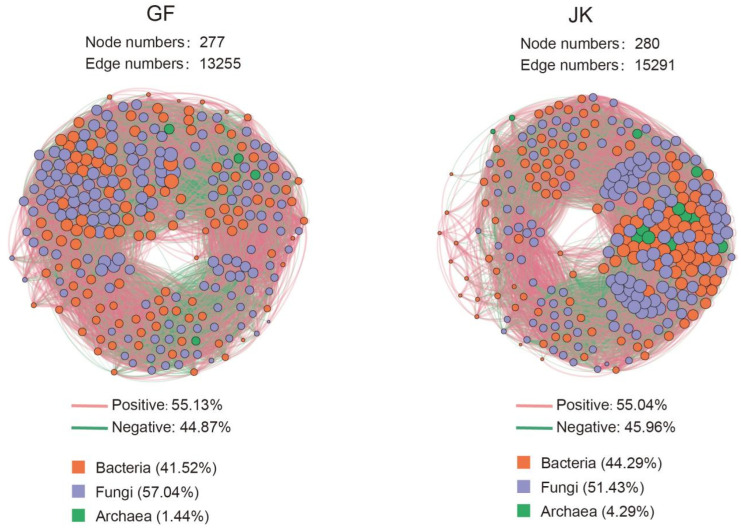
Cross-kingdom co-occurrence networks of rhizosphere microbial communities in healthy (JK) and root rot-diseased (GF) PKC. Node colors represent different kingdoms (orange: Bacteria, green: Fungi, purple: Archaea); edge colors represent correlation types (orange: positive, purple: negative). The healthy network is fungus-dominated (57.04% fungi), while the diseased network shifts to bacterium-dominated (55.71% bacteria), indicating fundamental network reorganization associated with root rot. Topological parameters are described in the main text. Networks are constructed based on strong and significant correlations (Spearman’s |ρ| > 0.6, *p* < 0.01, FDR-corrected). Abbreviations: JK, healthy plants; GF, root rot-affected (diseased) plants. Percentages may not sum to exactly 100% due to rounding.

To explore differences in rhizosphere microbial interaction patterns between healthy and root rot-affected PKC, we constructed cross-kingdom co-occurrence networks for healthy and diseased groups, respectively (Figure 5) [45]. Network topology parameters showed that the number of nodes (277 vs. 280) and edges (13,255 vs. 15,291) in the diseased network were higher than in the healthy network, indicating greater microbial interaction network complexity under disease conditions.

Strikingly, the positive correlation rate in the healthy network was 55.13%, slightly higher than the 55.04% in the diseased network; correspondingly, the negative correlation ratio increased from 44.87% to 45.96%, suggesting that disease may intensify competitive interactions within the microbial community. More importantly, there was a fundamental structural shift in network composition: the healthy network had the highest proportion of fungi (57.04%), exhibiting a fungus-dominated network characteristic. In the diseased network, bacteria became the dominant group (55.71%), with their relative abundance increasing from 41.52% to 44.29%, and archaea increasing from 1.44% to 4.29%. In summary, the networks of healthy and diseased plants differ markedly in multiple aspects: diseased networks exhibit greater complexity (more nodes and edges), higher negative correlation ratios (indicating intensified competition), and a fundamental kingdom-level shift from fungal to bacterial dominance. This ecological niche transition from fungal dominance to bacterial dominance reveals a fundamental reorganization of rhizosphere microbial communities during root rot disease progression, suggesting that pathogen infection may disrupt the original fungal network skeleton, thereby triggering bacterial community expansion and interaction network restructuring.

### 3.6. Fungal Trophic Mode Prediction Using FUNGuild

**Figure 6:**
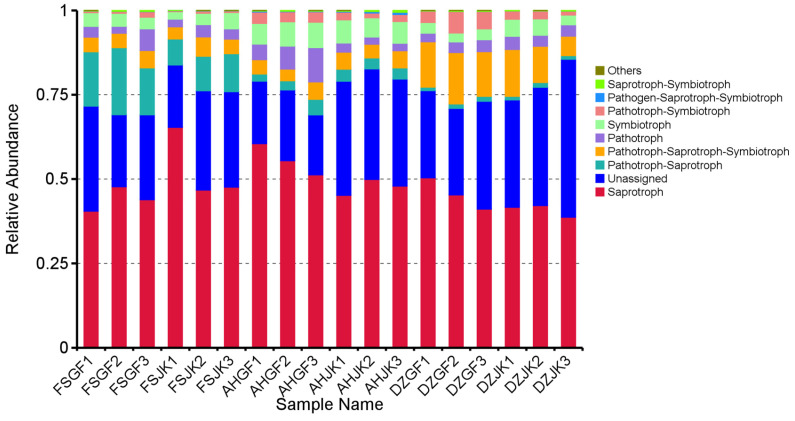
Relative abundance of fungal trophic modes in rhizosphere soils of healthy and root rot-affected PKC across three geographic locations. Abbreviations: FS, Lincang (Fengqing County); AH, Qujing (Huize County); DZ, Kunming (Xundian County); JK, healthy plants; GF, root rot-affected (diseased) plants.

Based on functional prediction analysis of fungal community trophic mode using the FUNGuild database (Figure 6) [41], the relative abundance of pathotrophic fungi in the rhizosphere soil of root rot-diseased plants (GF group) was consistently higher than that in healthy plants (JK group) across all three locations, while the relative abundance of symbiotrophic fungi consistently decreased in the diseased group. Saprotrophic fungi did not exhibit a consistent trend between healthy and diseased groups. These results indicate that root rot disease is associated with a shift in the PKC rhizosphere fungal community from a symbiotrophic/saprotrophic-dominated type toward a pathotroph-dominated type, suggesting a global functional reorganization of the fungal community in response to root rot. Notably, at the finer guild level (e.g., Plant_Pathogen, Arbuscular_Mycorrhizal), no individual guild showed statistically significant differences after FDR correction (Supplementary Table S3), indicating that disease-induced functional alterations are more pronounced at broader trophic categories than at the finer guild level. This pattern is consistently observed across all three locations (Supplementary Table S3), reinforcing the view that the functional shift is systemic rather than driven by a single guild.

### 3.7. Correlations Between Microbial Communities and Soil Environmental Factors

**Figure 7:**
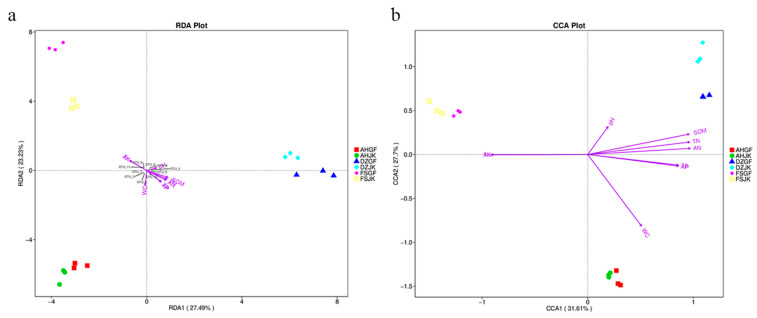
Redundancy analysis (RDA) and canonical correspondence analysis (CCA) of rhizosphere bacterial and fungal community structures with soil physicochemical properties in PKC. Abbreviations: FS, Lincang (Fengqing County); AH, Qujing (Huize County); DZ, Kunming (Xundian County); JK, healthy plants; GF, root rot-affected (diseased) plants. Note: (**a**) RDA plot of bacterial (16S rRNA) communities. RDA1 and RDA2 explained 27.49% and 23.23% of the total variance, respectively. (**b**) CCA plot of fungal (ITS) communities. CCA1 and CCA2 explained 31.61% and 27.7% of the total variance, respectively.

To examine the associations between soil physicochemical factors and the rhizosphere bacterial and fungal community structure of PKC, we conducted redundancy analysis (RDA) and canonical correspondence analysis (CCA), respectively (Figure 7a,b) [46]. RDA1 and RDA2 explained 27.49% and 23.23% of the total variation in bacterial communities, while CCA1 and CCA2 explained 31.61% and 27.7% of the total variation in fungal communities. Environmental factor fitting analysis (envfit) showed that, except for pH, all measured soil properties (WC, SOM, TN, TP, TK, AN, AP, AK) were significantly correlated with both bacterial and fungal community structure (*p* < 0.01).

To further verify the association between overall soil physicochemical factors and microbial communities, we conducted Mantel tests (based on Spearman rank correlation, 999 permutations) [38]. The results showed that the soil physicochemical property distance matrix (based on all nine factors) was significantly positively correlated with the bacterial community Bray–Curtis distance matrix (r = 0.725, *p* = 0.001) and also significantly positively correlated with the fungal community distance matrix (r = 0.768, *p* = 0.001) (Table 2). When extended to genus-level abundance, Mantel tests also revealed significant correlations for both bacteria (r = 0.612, *p* = 0.001) and fungi (r = 0.658, *p* = 0.001). Furthermore, soil properties were significantly correlated with alpha diversity indices: for bacteria, Chao1 (r = 0.487, *p* = 0.003) and Shannon (r = 0.523, *p* = 0.001); for fungi, Chao1 (r = 0.441, *p* = 0.008) and Shannon (r = 0.506, *p* = 0.001). These results indicate that soil physicochemical properties are associated with not only community composition but also species richness and diversity.

Spearman correlation analysis further screened key microbial genera significantly correlated with soil physicochemical factors (Supplementary Tables S1 and S2; the corresponding correlation heatmap is shown in Supplementary Figure S2) [47]. Among these, *Bradyrhizobium* was strongly positively correlated with SOM, TN, and AN (ρ > 0.80), while *Fusarium* was strongly negatively correlated with SOM and TN (ρ < −0.90). Other notable correlations included: *Rhodanobacter* showed strong negative correlations with SOM, TN, and AN (ρ < −0.70); *Acidiferrimicrobium* was positively correlated with WC (ρ = 0.59) and negatively with SOM and TN (ρ < −0.60); *Burkholderia-Caballeronia-Paraburkholderia* exhibited negative correlations with SOM, TN, and AN (ρ < −0.65); *Aspergillus* showed moderate negative correlations with multiple nutrients (ρ < −0.65); *Mortierella* was positively correlated with TK and negatively correlated with SOM and TN (ρ > 0.50); and *Meyerozyma* exhibited strong negative correlations with SOM, TN, and AN (ρ < −0.85). From the sample distribution in the ordination plots, samples from different geographical locations (AH, DZ, FS) were clearly separated along the main axes, while healthy and diseased samples from the same location were intermingled, showing no separation trend. Together, these results demonstrate that geographical location and soil physicochemical properties are the dominant factors associated with the structure of both bacterial and fungal communities in the PKC rhizosphere, being more strongly associated with plant health status.

## 4. Discussion

Consistent with previous large-scale studies [11,17], our results indicate that geographical origin was the strongest correlate of rhizosphere microbial community variation in PKC, while plant health status exerts a comparatively weaker, site-specific effect. This finding aligns with the view that soil microbial communities are mainly structured by environmental factors at larger spatial scales [8]. A recent study on root rot of *Knoxia roxburghii* similarly confirmed that geographic location and soil properties, rather than disease status alone, are associated with rhizosphere microbial community variation [48]. Significant differences in soil physicochemical properties such as organic matter, total nitrogen, and total potassium were observed among the three locations, but differences between healthy and diseased plants were not uniform. The water content of diseased soil in Lincang significantly increased, while organic matter and nitrogen content significantly decreased; Kunming showed only an increase in water content; and Qujing showed no significant differences in any of the nine measured indicators between healthy and diseased plants. This indicates that root rot occurrence is not necessarily accompanied by systematic changes in soil nutrients, reinforcing the view that disease in different locations may be linked to distinct microecological correlates rather than a universal nutrient-deficiency pattern [4,9]. Alpha and beta diversity analyses further showed that microbial community structures were significantly separated by location, while healthy and diseased samples within the same location were intermingled, once again highlighting the strong association between geographical environment and microbial community structure [8].

Notably, despite strong geographic signatures, we identified conserved microbial shifts across all three locations. LEfSe analysis identified that *Acidiferrimicrobium* and HSB_OF53_F07 were consistently enriched in healthy groups, while *Burkholderia-Caballeronia-Paraburkholderia* and *Rhodanobacter* were enriched in diseased groups [44]. A parallel study on grapevine root rot demonstrated that healthy plants recruit core beneficial bacteria (*Pseudomonas*, *Bacillus*, and *Streptomyces*) from the rhizosphere, which enhance disease resistance [49]. These cross-location consistent enrichment patterns suggest they may serve as potential biological indicators for PKC rhizosphere health.

Importantly, the disease-associated bacteria identified by LEfSe—*Burkholderia-Caballeronia-Paraburkholderia* and *Rhodanobacter*—consistently co-occur with *Fusarium* across all three locations, suggesting they form part of a multi-kingdom pathobiome rather than acting as independent pathogens. These bacterial taxa may contribute to disease progression by facilitating fungal infection through siderophore production, degradation of plant defense compounds, or exploitation of root tissues damaged by *Fusarium* [8]. Conversely, the health-enriched genera *Acidiferrimicrobium* and HSB_OF53_F07 are likely components of the native disease-suppressive microbiome, and their decline may weaken the soil’s natural resistance to pathogen invasion. Importantly, the composition and activity of this pathobiome are not fixed but are associated with soil properties: in nutrient-rich Kunming soils, the pathobiome exhibited more complex network reorganization, whereas in Qujing—where minimal nutrient differences were observed between healthy and diseased soils—the pathobiome shifts were more subtle and primarily functional (trophic mode changes) rather than compositional. This supports the view that the pathobiome is a dynamic consortium influenced by both biotic interactions and the edaphic environment.

Unlike the commonly held belief that *Fusarium* is the main pathogen of root rot [3,20], *Fusarium* was highly enriched in all samples and showed no significant differences between healthy and diseased individuals. Using integrated multiomics, Pang et al. [20] systematically studied *Polygonatum cyrtonema* rhizome rot and found that although *Fusarium oxysporum* and *Colletotrichum spaethianum* were confirmed as pathogens, healthy rhizosphere soil was enriched with beneficial *Streptomyces* that could suppress root rot. This suggests that *Fusarium* may be a resident core microbiota in the PKC rhizosphere, only transforming into a pathogenic state under specific conditions, consistent with the observations of Gordon [50] that many *Fusarium* species are opportunistic pathogens.

More importantly, FUNGuild functional prediction revealed a common functional transformation across all three locations: the relative abundance of pathotrophic fungi in diseased plant rhizosphere was consistently higher than in healthy plants, while symbiotrophic fungi were consistently reduced [41]. Although FUNGuild analysis did not identify individual guilds that reached statistical significance after FDR correction (q < 0.05, Supplementary Table S3), the consistent shift at the trophic mode level-increased pathotrophs and decreased symbiotrophs-points to a global nutritional reorganization of the fungal community in response to root rot. This aligns with findings from *Taraxacum kok-saghyz* root rot, where diseased plants showed significant enrichment of potential pathogenic taxa including *Aspergillus*, *Fusarium*, *Alternaria*, and *Paramyrothecium*, while beneficial taxa including *Sphingomonas*, *Gemmatimonas*, and Bacillus were predominantly enriched in healthy rhizosphere [51]. This suggests that disease-induced functional alterations may be more pronounced at broader trophic categories than at the finer guild level [10,16]. The observation that pathotrophs increase while symbiotrophs decrease across all three locations, despite the absence of significant changes in any single guild, implies that the overall functional shift is driven by coordinated changes across multiple pathotrophic and symbiotrophic lineages rather than by the expansion of a single dominant guild.

The cross-kingdom reconstruction of the rhizosphere microbial interaction network represents another important discovery. The healthy network was dominated by fungi (57.04%), while bacteria became the dominant group in the diseased network (55.71%), showing an ecological niche transition from fungal dominance to bacterial dominance [45]. Simultaneously, the negative correlation rate in the diseased network increased from 44.87% to 45.96%, indicating that disease may intensify competitive interactions within the microbial community [52]. A recent study on tobacco root rot caused by *Fusarium solani* revealed a strikingly similar pattern: diseased plants exhibited systematic reorganization of cross-kingdom co-occurrence networks, with bacterial-fungal interactions decreasing by 8–27% compared to healthy plants [52]. This type of network reconstruction has not been previously reported in studies on PKC root rot, suggesting that pathogen infection may disrupt the original fungal network skeleton, thereby triggering bacterial community expansion and interaction relationship reorganization.

Mechanistically, this striking ecological shift from fungal dominance to bacterial dominance may be driven by pathogen-induced alterations in root exudation patterns and tissue permeability [13]. In healthy plants, balanced root exudation—comprising sugars, organic acids, and secondary metabolites—likely maintains a stable, fungus-dominated community by providing consistent carbon sources that support symbiotic fungi and beneficial bacteria such as *Acidiferrimicrobium*. Upon pathogen infection, however, root tissues become more permeable due to cell wall degradation by fungal enzymes (e.g., cellulases and pectinases), leading to increased leakage of intracellular nutrients into the rhizosphere. This nutrient spillover creates a “hotspot” that favors fast-growing, copiotrophic bacteria (e.g., *Burkholderia* and *Rhodanobacter*) over slower-growing fungi, which is consistent with our observed network shift from fungal to bacterial dominance in diseased soils. Concurrently, infection may suppress the secretion of defense-related secondary metabolites that normally inhibit opportunistic pathogens, further facilitating *Fusarium* proliferation. Several additional ecological mechanisms may also contribute to this reorganization. First, root rot pathogens may directly suppress or kill specific fungal taxa that serve as network hubs, creating vacant niches that are rapidly occupied by opportunistic bacteria. Second, infection-induced changes in root exudate composition-such as altered ratios of sugars, amino acids, and organic acids-may differentially affect fungal and bacterial growth, favoring fast-growing bacterial opportunists over slower-growing fungi. Third, the increased proportion of negative correlations in the diseased network suggests intensified resource competition, potentially driven by pathogen-induced nutrient limitation or niche contraction.

RDA and CCA analyses showed that, except for pH, all soil physicochemical factors were significantly correlated with microbial community structure [46]. Samples were strictly sorted by geographical origin, with no separation between healthy and diseased states, once again indicating that geographically related soil heterogeneity is strongly associated with microbial community structure [48]. This result underscores a critical methodological implication: when conducting comparative studies on diseases, it is necessary to carefully control for differences in geographical and soil backgrounds; otherwise, geographical signals may be mistaken for disease signals. The Mantel test results (bacteria: r = 0.725, *p* = 0.001; fungi: r = 0.768, *p* = 0.001) independently corroborated the RDA/CCA findings, indicating that soil physicochemical properties are significant correlates of rhizosphere microbial community assembly [40]. Notably, the higher correlation coefficient for fungi (r = 0.768 vs. 0.725 for bacteria) suggests that fungal communities may be more responsive to edaphic variations than bacterial communities, potentially due to their greater sensitivity to moisture and organic matter fluctuations.

Several limitations of this study should be acknowledged. Our amplicon sequencing approach provides relative abundance information with limited taxonomic resolution, and the single time-point sampling cannot capture the temporal dynamics of microbial shifts. Moreover, although we proposed mechanistic hypotheses regarding root exudates and tissue permeability, these variables were not directly measured in the present study. Future research integrating root exudate metabolomics, tissue permeability assays, metagenomics, culturomics, and longitudinal sampling would help to functionally validate the roles of the identified indicator genera and directly test the mechanistic hypotheses proposed here. As reviewed recently, rhizosphere engineering approaches-including the deployment of synthetic microbial communities (SynComs) and root exudate manipulation-hold promise for translating microbiome discoveries into sustainable crop protection strategies [53].

Despite these limitations, the above results systematically compare the rhizosphere microbiota of PKC from three geographical locations for the first time. The core novelties of this study lie in: (i) identifying cross-location consistent bacterial indicator genera (healthy-enriched *Acidiferrimicrobium* and HSB_OF53_F07, diseased-enriched *Burkholderia-Caballeronia-Paraburkholderia* and *Rhodanobacter*); (ii) revealing increased pathotrophic fungi and decreased symbiotrophic fungi as common functional changes across all three locations; (iii) providing the first report of an ecological niche transition in the rhizosphere microbial network from fungal dominance to bacterial dominance induced by root rot disease; and (iv) proposing a mechanistic framework linking pathogen-induced root permeability changes to the observed kingdom-level network reorganization. These findings provide new insights into the microecological mechanisms of PKC root rot and offer a theoretical basis for green prevention and control strategies such as targeted restoration of symbiotic fungi and regulation of bacterial indicator genera.

## 5. Conclusions

In conclusion, this study provides the first systematic evidence of location-transcending microbial dynamics associated with PKC root rot in Yunnan Province. Four bacterial genera (*Acidiferrimicrobium*, HSB_OF53_F07, *Burkholderia-Caballeronia-Paraburkholderia*, and *Rhodanobacter*) emerged as consistent indicators across three geographically distinct sites. Fungal trophic modes shifted predictably (pathotrophs ↑, symbiotrophs ↓), and the rhizosphere network underwent a fundamental reorganization from fungus-dominated to bacterium-dominated. Nevertheless, geographic origin and soil physicochemical properties remained the strongest correlates of microbial community variation, underscoring the importance of soil background in disease studies. These findings establish a microecological framework for understanding PKC root rot and provide potential biomarkers for green disease management, such as targeted restoration of symbiotrophic fungi or modulation of specific bacterial indicator genera.

## Figures and Tables

**Table 1:** Physicochemical properties of rhizosphere soils from healthy and root rot-affected PKC across three locations in Yunnan.

	FSGF	FSJK	AHGF	AHJK	DZGF	DZJK
WC	28.03 ± 0.59 c	24.43 ± 0.19 d	41.41 ± 0.10 a	42.25 ± 0.27 a	37.41 ± 1.80 b	27.45 ± 0.76 c
SOM	22.47 ± 4.24 d	44.73 ± 2.46 c	98.72 ± 2.74 b	105.23 ± 3.77 b	182.12 ± 3.43 a	191.72 ± 6.68 a
TN	1.08 ± 0.08 d	2.70 ± 0.14 c	5.33 ± 0.16 b	5.25 ± 0.18 b	8.50 ± 0.17 a	8.38 ± 0.32 a
TP	0.54 ± 0.09 c	0.70 ± 0.08 bc	0.93 ± 0.16 ab	0.92 ± 0.12 ab	1.00 ± 0.08 a	1.03 ± 0.02 a
TK	13.33 ± 0.79 a	12.10 ± 0.40 a	10.29 ± 0.85 b	9.76 ± 0.70 bc	9.08 ± 0.58 bc	8.17 ± 0.23 c
AN	221.97 ± 34.91 d	424.88 ± 19.21 c	798.91 ± 25.31 b	774.85 ± 24.85 b	1112.12 ± 2.84 a	1118.93 ± 7.74 a
AP	68.56 ± 10.16 c	88.94 ± 9.91 bc	118.91 ± 19.90 ab	118.42 ± 15.48 ab	126.28 ± 9.01 a	131.93 ± 2.98 a
AK	148.31 ± 12.57 c	136.66 ± 5.00 bc	116.68 ± 9.99 ab	111.68 ± 5.00 ab	103.36 ± 5.77 a	95.03 ± 2.89 a
pH	7.20 ± 0.14 a	7.21 ± 0.12 a	7.16 ± 0.02 a	7.17 ± 0.05 a	7.30 ± 0.25 a	7.25 ± 0.07 a

Note: Different lowercase letters indicate significant differences among all six groups based on one-way ANOVA with Tukey’s HSD post hoc test (*p* < 0.05). These letters primarily reflect geographic variation. Paired comparisons between healthy and diseased groups within the same location were performed using independent-sample *t*-tests, and the corresponding significances are described in the text. Abbreviations: FS, Lincang (Fengqing County); AH, Qujing (Huize County); DZ, Kunming (Xundian County); JK, healthy plants; GF, root rot-affected (diseased) plants.

**Table 2:** Mantel test correlations between soil physicochemical properties and microbial communities.

Comparison	r	*p*-Value	Permutations
Soil properties vs. bacterial community composition	0.725	0.001	999
Soil properties vs. fungal community composition	0.768	0.001	999
Soil properties vs. bacterial genus-level composition	0.612	0.001	999
Soil properties vs. fungal genus-level composition	0.658	0.001	999
Soil properties vs. bacterial Chao1	0.487	0.003	999
Soil properties vs. bacterial Shannon	0.523	0.001	999
Soil properties vs. fungal Chao1	0.441	0.008	999
Soil properties vs. fungal Shannon	0.506	0.001	999

Note: Mantel tests were performed using Spearman’s rank correlation based on Euclidean distance of nine soil physicochemical properties (WC, SOM, TN, TP, TK, AN, AP, AK, pH) and Bray–Curtis distance for bacteria/weighted UniFrac distance for fungi.

## Data Availability

The raw sequencing data have been deposited in the NCBI Sequence Read Archive (SRA) under BioProject accession number PRJNA1475215.

## Supplementary Materials

The following supporting information can be downloaded at: https://www.mdpi.com/article/10.3390/microorganisms14071568/s1, Figure S1: Morphological comparison of healthy and root rot-affected *Polygonatum kingianum* Coll. (PKC) plants; Table S1: Spearman rank correlation coefficients between soil physicochemical properties and the relative abundance of bacterial genera (mean relative abundance > 0.1%); Table S2: Spearman rank correlation coefficients between soil physicochemical properties and the relative abundance of fungal genera (mean relative abundance > 0.1%); Figure S2: Spearman correlation heatmap between soil physicochemical properties and (A) bacterial genera, (B) fungal genera; Table S3: FUNGuild-based functional guild abundances in rhizosphere fungal communities (no significant differences detected at q < 0.05); Table S4: Differentially abundant KEGG pathways (q < 0.05) in rhizosphere bacterial communities of Lincang (FS) samples; Table S5: Differentially abundant KEGG pathways (q < 0.05) in rhizosphere bacterial communities of Kunming (DZ) samples; Figure S3: Heatmap of differentially abundant KEGG pathways (q < 0.05) in Lincang (FS) rhizosphere bacterial communities.

## Abbreviations

The following abbreviations are used in this manuscript:

PKC	*Polygonatum kingianum* Coll.
WC	water content
TP	total phosphorus
TK	total potassium
TN	total nitrogen
SOM	soil organic matter
AN	alkaline nitrogen
AK	available potassium
AP	available phosphorus
RDA	redundancy analysis
CCA	canonical correspondence analysis
FDR	false discovery rate
ITS	internal transcribed spacer
KEGG	Kyoto Encyclopedia of Genes and Genomes
LDA	linear discriminant analysis
LEfSe	linear discriminant analysis effect size
OTU	operational taxonomic unit
PCoA	principal coordinate analysis
PERMANOVA	permutational multivariate analysis of variance

## References

[1] Zhao M., Jia H., Zhao J., Wang Y., Laraib I., Shi X., Hao J., Chu Q. (2025). Response of cultivation suitability for *Polygonatum kingianum* to climate change in China. Environ. Earth Sci..

[2] Yang L., Yang Q., Wulu J., Wang Y., Jin W., Yan Z., Zhang Z. (2024). Quality analysis and function prediction of soil microbial communities of *Polygonatum cyrtonema* in two indigenous-origins. Front. Microbiol..

[3] Liu R., Chen Y., Wu W., Wang R., Cheng Y., Lu P., Yang L., Dai Y. (2026). Identification of root rot pathogens, biological control, and the mechanism analysis underlying *Polygonatum kingianum* response to pathogen stress. Plant Dis..

[4] Xu X., Wan J., Liu G., Lu C., Mao X., Wu J., Liu H., Ding Y., Xu P. (2025). Physiological, transcriptomic and metabolomic analyses reveal the mechanism of CuO and silicon nanoparticles involved in *Polygonatum kingianum* response to root rot. Chem. Biol. Technol. Agric..

[5] Vayssier-Taussat M., Albina E., Citti C., Cosson J.F., Jacques M.A., Lebrun M.H., Le Loir Y., Ogliastro M., Petit M.A., Roumagnac P. (2014). Shifting the paradigm from pathogens to pathobiome: New concepts in the light of meta-omics. Front. Cell. Infect. Microbiol..

[6] Trivedi P., Leach J.E., Tringe S.G., Sa T., Singh B.K. (2020). Plant–microbiome interactions: From community assembly to plant health. Nat. Rev. Microbiol..

[7] Sırcan A.K., Streck T., Schnepf A., Giraud M., Lattacher A., Kandeler E., Poll C., Pagel H. (2025). Trait-based modeling of microbial interactions and carbon turnover in the rhizosphere. Soil Biol. Biochem..

[8] Mendes R., Garbeva P., Raaijmakers J.M. (2013). The rhizosphere microbiome: Significance of plant beneficial, plant pathogenic, and human pathogenic microorganisms. FEMS Microbiol. Rev..

[9] Nishisaka C.S., Quevedo H.D., Ventura J.P., Andreote F.D., Mauchline T.H., Mendes R. (2025). Soil microbial diversity: A key factor in pathogen suppression and inoculant performance. Geoderma.

[10] Frąc M., Hannula S.E., Bełka M., Jedryczka M. (2018). Fungal biodiversity and their role in soil health. Front. Microbiol..

[11] Raaijmakers J.M., Paulitz T.C., Steinberg C., Alabouvette C., Loccoz Y.M. (2009). The rhizosphere: A playground and battlefield for soilborne pathogens and beneficial microorganisms. Plant Soil.

[12] Aguilar-Trigueros C.A., Powell J.R., Anderson I.C., Antonovics J., Rilling M.C. (2014). Ecological understanding of root-infecting fungi using trait-based approaches. Trends Plant Sci..

[13] Gamliel A., Austerweil M., Kritzman G. (2000). Non-chemical approach to soilborne pest management–organic amendments. Crop Prot..

[14] Tilman D., Reich P.B., Knops J.M.H. (2006). Biodiversity and ecosystem stability in a decade-long grassland experiment. Nature.

[15] Han Y., Xu L., Liu L., Yi M., Guo E., Zhang A., Yi H. (2017). Illumina sequencing reveals a rhizosphere bacterial community associated with foxtail millet smut disease suppression. Plant Soil.

[16] Pandey S.N., Abid M., Abid Ali Khan M.M. (2018). Diversity, functions, and stress responses of soil microorganisms. Plant Microbiome: Stress Response.

[17] Batista B.D., Wang J., Liu H., Kaur S., Macdonald C.A., Qiu Z., Trivedi P., Delgado-Baquerizo M., Xiong C., Liang J. (2024). Biotic and abiotic responses to soilborne pathogens and environmental predictors of soil health. Soil Biol. Biochem..

[18] Pang Z., Mao X., Xia Y., Xiao J., Wang X., Xu P., Liu G. (2022). Multiomics reveals the effect of root rot on *Polygonatum rhizome* and identifies pathogens and biocontrol strain. Microbiol. Spectr..

[19] Liu J., Qian Y., Yang W., Yang M., Zhang Y., Duan B., Yang Y., Tao A., Xia C. (2024). Elucidating the interaction of rhizosphere microorganisms and environmental factors influencing the quality of *Polygonatum kingianum* Coll. et Hemsl. Sci. Rep..

[20] Ji L., Nasir F., Tian L., Chang J.J., Sun Y., Zhang J.F., Li X.J., Tian C.J. (2021). Outbreaks of root rot disease in different aged American *Ginseng* plants are associated with field microbial dynamics. Front. Microbiol..

[21] Najdenko E., Lorenz F., Dittert K., Olfs H. (2024). Rapid in-field soil analysis of plant-available nutrients and pH for precision agriculture-A review. Precis. Agric..

[22] Shigaki F., Sharpley A. (2011). Phosphorus source and soil properties effects on phosphorus availability. Soil Sci..

[23] Du E., Xia N., Tang Y., Guo Z., Guo Y., Wang Y., Viries W. (2022). Anthropogenic and climatic shaping of soil nitrogen properties across urban-rural-natural forests in the Beijing metropolitan region. Geoderma.

[24] Lu D.J., Li C.Z., Sokolwski E., Magen H., Chen X.Q., Wang H.Y., Zhou J.M. (2017). Crop yield and soil available potassium changes as affected by potassium rate in rice-wheat systems. Field Crops Res..

[25] Wang Z., Yang W., Bai Y., Song Y., Li M., Sun L. (2025). Development of portable soil organic matter system based image and spectral fusion. Meas. Sci. Technol..

[26] Zhang J., Wei L., Yang J., Ahmed W., Wang Y., Ji G. (2020). Probiotic consortia: Reshaping the rhizospheric microbiome and its role in suppressing root-rot disease of *Panax notoginseng*. Front. Microbiol..

[27] Gao H., Hua C., Tong M. (2018). Impact of Dinophysis acuminata feeding Mesodinium rubrum on nutrient dynamics and bacterial composition in a microcosm. Toxins.

[28] Bolger A.M., Lohse M., Usadel B. (2014). Trimmomatic: A flexible trimmer for Illumina sequence data. Bioinformatics.

[29] Magoč T., Salzberg S.L. (2011). FLASH: Fast length adjustment of short reads to improve genome assemblies. Bioinformatics.

[30] Edgar R.C., Haas B.J., Clemente J.C., Quince C., Knight R. (2011). UCHIME improves sensitivity and speed of chimera detection. Bioinformatics.

[31] Edgar R.C. (2013). UPARSE: Highly accurate OTU sequences from microbial amplicon reads. Nat. Methods.

[32] Quast C., Pruesse E., Yilmaz P., Gerken J., Schweer T., Yarza P., Peplies J., Glöckner F.O. (2012). The SILVA ribosomal RNA gene database project: Improved data processing and web-based tools. Nucleic Acids Res..

[33] Abarenkov K., Nilsson R.H., Larsson K.H., Taylor A.F.S., May T.W., Frøslev T.G., Pawlowska J.P., Lindahl B., Põldmaa K., Truong C. (2024). The UNITE database for molecular identification and taxonomic communication of fungi and other eukaryotes: Sequences, taxa and classifications reconsidered. Nucleic Acids Res..

[34] Bolyen E., Rideout J.R., Dillon M.R., Bokulich N.A., Abnet C.C., Al-Ghalith G.A., Alexander H., Alm H., Arumugam M., Asnicar F. (2019). Reproducible, interactive, scalable and extensible microbiome data science using QIIME 2. Nat. Biotechnol..

[35] Barnett D., Arts I., Penders J. (2021). microViz: An R package for microbiome data visualization and statistics. J. Open Source Softw..

[36] Segata N., Izard J., Waldron L., Gevers D., Miropolsky L., Garrett W.S., Huttenhower C. (2011). Metagenomic biomarker discovery and explanation. Genome Biol..

[37] Bastian M., Heymann S., Jacomy M. (2009). Gephi: An open source software for exploring and manipulating networks. Proc. Int. AAAI Conf. Web Soc. Media.

[38] Mantel N. (1967). The detection of disease clustering and a generalized regression approach. Cancer Res..

[39] Kanduri C., Maria M., Olstad E.W., Zucknick M., Li J.J., Sandve G.K. (2025). Beware of counter-intuitive levels of false discoveries in datasets with strong intra-correlations. Genome Biol..

[40] Schumacher S.M., Doyle W.J., Hill K., Ochoa-Repáraz J. (2025). PICRUSt2 analysis of fecal microbiome associated with a murine model of multiple sclerosis. FASEB Bioadv..

[41] Nguyen N.H., Song Z., Bates S.T., Branco S., Tedersoo L., Menke J., Schilling J.S., Kennedy P.G. (2016). FUNGuild: An open annotation tool for parsing fungal community datasets by ecological guild. Fungal Ecol..

[42] Wilcoxon F. (1945). Individual comparisons by ranking methods. Biom. Bull..

[43] Gower J.C. (1966). Some distance properties of latent root and vector methods used in multivariate analysis. Biometrika.

[44] Pang S. (2025). Improving linear discriminant analysis effect size analysis to enhance its reliability in small sample sizes. Turk. J. Pediatr..

[45] Ding Y., Liu M., Zhang N., Zhang F., Yang X., Yuan J. (2026). *Stellera chamaejasme* L. expansion-induced dynamic reorganization of soil microbial co-occurrence networks and their topological properties in subalpine meadows. J. Environ. Manag..

[46] Li Z., He J., Shen J., Li Y., Yuan Q., Zhu Q., Wu J. (2025). Origin and assembly characteristics of periphyton microbes in subtropical paddy fields. Appl. Soil Ecol..

[47] Zhang Q. (2025). On relationships between Chatterjee’s and Spearman’s correlation coefficients. Commun. Stat. Theory Methods.

[48] Liu C., Li H., Dong J., He X., Zhang L., Qiu B. (2024). Structure and function of rhizosphere soil microbial communities associated with root rot of *Knoxia roxburghii*. Front. Microbiol..

[49] Wang R., Zhang W., He Z., Zhou Y., Chen C., Song K., Shang Q., Wu Y., Gu P., Shu D. (2026). Core microbiota recruited by healthy grapevines enhance resistance against root rot disease. Genome Biol..

[50] Gordon T.R. (2017). *Fusarium oxysporum* and the *Fusarium wilt* syndrome. Annu. Rev. Phytopathol..

[51] Nie Q., Ao G., Wang S., Deng T., Zhou H., Wang M., Wang X. (2025). Disease severity of root rot affects rhizosphere microbial community structure and network stability of *Taraxacum kok-saghyz* Rodin. Rhizosphere.

[52] Zhao W., Liang H., Liu K., Wang J., Wang Z., Lu J., Zhao W., Wang L., Yang J., Zhang Y. (2026). Shifts in rhizosphere microbial cross-kingdom co-occurrence network underpin *Fusarium solani*-mediated root rot in tobacco. Ind. Crops Prod..

[53] Agorsor I.D.K. (2026). Rhizosphere engineering for improved plant-beneficial microbe interactions: Concepts and some remaining questions. Curr. Plant Biol..

