# Supplementary material for: Rhizosphere Microbiome Dynamics Associated with Root Rot in Polygonatum kingianum Coll
Source: Microorganisms. 2026 Jul 17;14(7):1568. doi: 10.3390/microorganisms14071568 (PMC13413765; doi:10.3390/microorganisms14071568)

Supplementary Materials

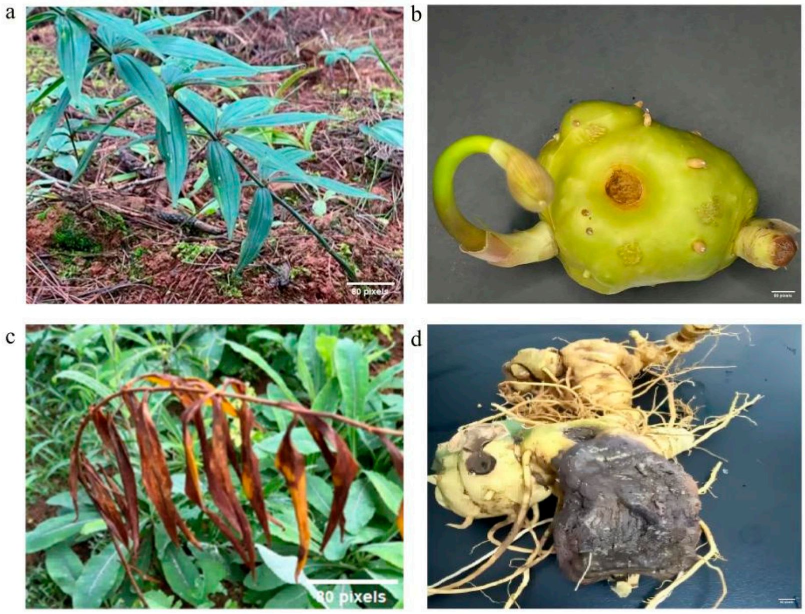

Figure S1. Morphological comparison of healthy and root rot-affected *Polygonatum kingianum* Coll. (PKC) plants. (a) Above-ground parts of a healthy plant; (b) below-ground parts (rhizomes) of a healthy plant; (c) above-ground parts of a diseased plant showing wilting and leaf yellowing; (d) below-ground parts (rhizomes) of a diseased plant showing browning, necrosis, water-soaked lesions, and soft rot. Scale bars are approximate.

Table S1. Spearman rank correlation coefficients between soil physicochemical properties and the relative abundance of bacterial genera at the genus level.

Notes: Only genera with mean relative abundance > 0.1% across all samples and at least one significant correlation (FDR-adjusted $p < 0.05$) with $|\rho| > 0.6$ are shown. Bold values indicate strong correlations ($|\rho| > 0.7$). The complete correlation matrix including all 49 bacterial genera is available upon request. FDR correction was applied to control for multiple comparisons (Benjamini-Hochberg method). Abbreviations: WC, water content; SOM, soil organic matter; TN, total nitrogen; TP, total phosphorus; TK, total potassium; AN, alkaline nitrogen; AP, available phosphorus; AK, available potassium.

Table S2. Spearman rank correlation coefficients between soil physicochemical properties and the relative abundance of fungal genera at the genus level.

Notes: Only genera with mean relative abundance > 0.1% across all samples and at least one significant correlation (FDR-adjusted $p < 0.05$) with $|\rho| > 0.6$ are shown. Bold values indicate strong correlations ($|\rho| > 0.7$). The complete correlation matrix

including all 50 fungal genera is available upon request. FDR correction was applied to control for multiple comparisons (Benjamini-Hochberg method). Abbreviations: WC, water content; SOM, soil organic matter; TN, total nitrogen; TP, total phosphorus; TK, total potassium; AN, alkaline nitrogen; AP, available phosphorus; AK, available potassium.

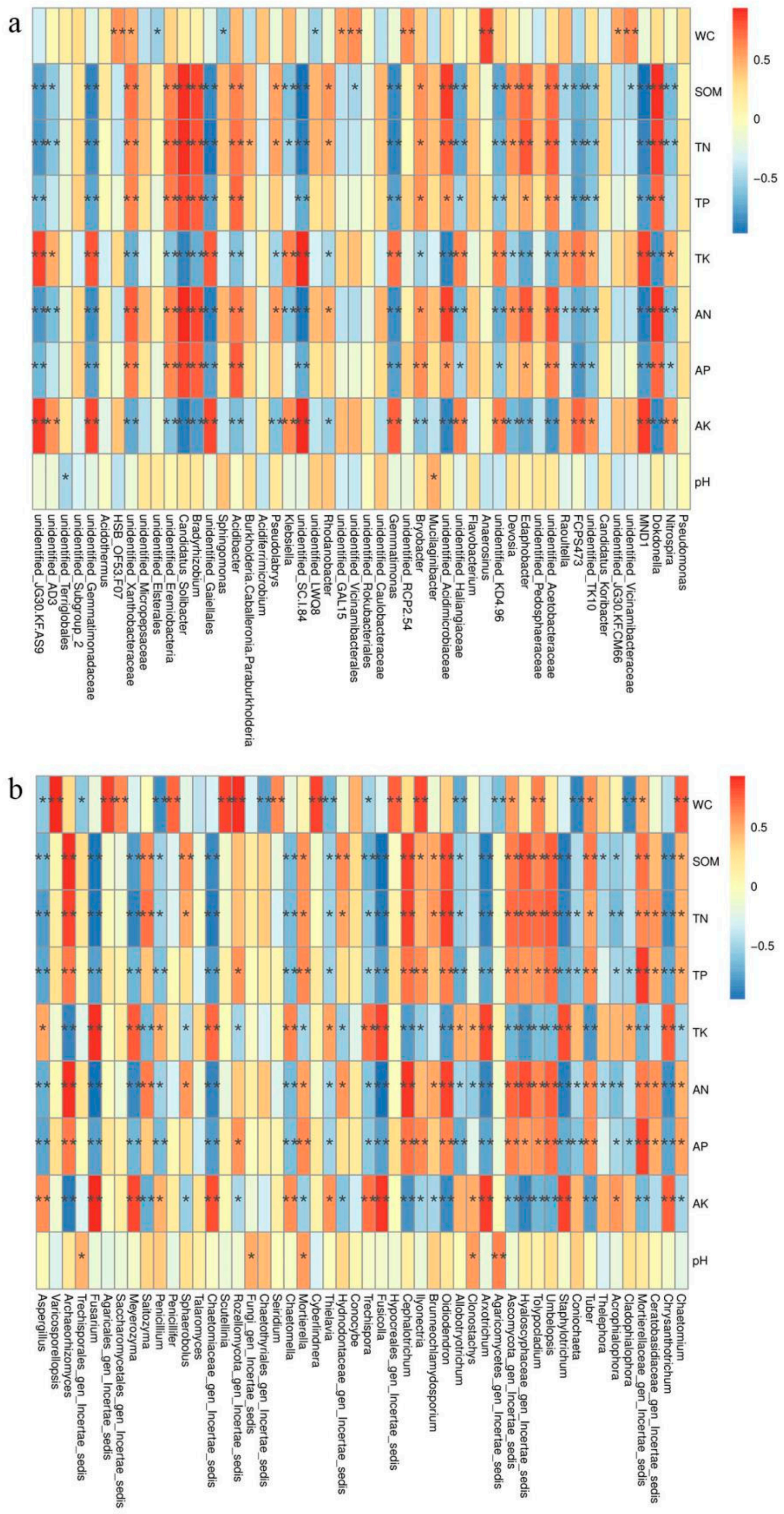

Figure S2. Spearman correlation heatmap between soil physicochemical properties

and (A) bacterial genera, (B) fungal genera. Red and blue colors indicate positive and negative correlations, respectively. Only genera with mean relative abundance > 0.1% are shown. *FDR-adjusted p < 0.05; **FDR-adjusted p < 0.01; ***FDR-adjusted p < 0.001. Abbreviations: WC, water content; SOM, soil organic matter; TN, total nitrogen; TP, total phosphorus; TK, total potassium; AN, alkaline nitrogen; AP, available phosphorus; AK, available potassium.

Table S3. FUNGuild-based functional guild abundances in rhizosphere fungal communities (representative data from Lincang (FS); no significant differences detected at q < 0.05 at any location).

Guild	avg(FSGF)	avg(FSJK)	p-value	q-value
Arbuscular_Mycorrhizal	0.00859	0.00504	0.1000	0.525
Ericoid_Mycorrhizal	0.00039	0.00006	0.0765	0.525
Plant_Pathogen	0.03779	0.02849	1.0000	1.000
Animal_Pathogen-Plant_Pathogen-Undefined_Saprotroph	0.00507	0.00264	0.1000	0.525
Soil_Saprotroph	0.00755	0.00419	0.1000	0.525
Wood_Saprotroph	0.00388	0.02823	0.1000	0.525
Undefined_Saprotroph	0.42774	0.47406	0.7000	1.000

Notes: Trophic modes are summarized in Figure 6 of the main text. No guild showed statistically significant differences after FDR correction (q < 0.05) at any of the three locations. Data shown are from Lincang (FS) as a representative site. Results for Qujing (AH) and Kunming (DZ) are consistent (no significant guilds) and are available upon request. This lack of significance at the guild level, despite the consistent shift at the broader trophic mode level (Figure 6), suggests that the overall functional transition from symbiotrophy to pathotrophy is driven by coordinated changes across multiple guilds rather than by the expansion of a single dominant pathogenic guild. FDR correction was applied using the Benjamini-Hochberg method. Given the small sample size (n = 3 per group), the non-parametric Wilcoxon rank-sum test has limited power to detect subtle differences at the guild level (the minimum attainable two-sided p-value is 0.1). The specific p-value of 1.000 observed for certain guilds (e.g., Plant_Pathogen) indicates that the ranks of relative abundances were completely overlapping between the two groups, suggesting no detectable difference in that particular guild at the observational level. This does not contradict the overall functional shift observed at the broader trophic mode level, which likely results from the cumulative effects of multiple guild-level changes that are too small to reach significance individually.

Table S4. Differentially abundant KEGG pathways (q < 0.05) in rhizosphere bacterial communities of Lincang (FS) between healthy (FSJK) and root rot-affected (FSGF) samples (representative selection).

Pathway ID	Pathway Name	avg(FSGF)	avg(FSJK)	log2FC	p-value	q-value
PWY-7094	ABC transporters	0.00718	0.00590	0.28	0.00027	0.00047
FASYN-ELONG-PWY	Fatty acid biosynthesis elongation	0.00702	0.00649	0.11	0.00073	0.00061
PWY-6901	Putative stress response pathway	0.00143	0.00129	0.15	0.22643	0.01607
BRANCHED-CHAIN-AA-SYN-PWY	Branched-chain amino acid biosynthesis	0.00861	0.00911	-0.08	0.00037	0.00047
TCA	TCA cycle	0.00742	0.00750	-0.02	0.00159	0.00094
DAPLYSINESYN-PWY	Lysine biosynthesis	0.00501	0.00545	-0.12	0.00018	0.03369

Notes: log2FC: positive values indicate higher abundance in diseased samples (FSGF); negative values indicate higher abundance in healthy samples (FSJK). FDR correction was applied using the Benjamini-Hochberg method. Only representative pathways with significant differences ($q < 0.05$) are shown. The complete list of 102 significant pathways is available upon request.

Table S5. Differentially abundant KEGG pathways ($q < 0.05$) in rhizosphere bacterial communities of Kunming (DZ) between healthy (DZJK) and root rot-affected (DZGF) samples.

Pathway ID	avg(DZGF)	avg(DZJK)	log2FC	p-value	q-value
DAPLYSINESYN-PWY	0.00540	0.00554	-0.04	0.00018	0.03369
PWY-6901	0.00162	0.00112	0.53	0.00039	0.03369
P122-PWY	0.00090	0.00058	0.63	0.00032	0.03369

Notes: log2FC: positive values indicate higher abundance in diseased samples (DZGF); negative values indicate higher abundance in healthy samples (DZJK). FDR correction was applied using the Benjamini-Hochberg method.

Figure S3. Heatmap of differentially abundant KEGG pathways ($q < 0.05$) in Lincang (FS) rhizosphere bacterial communities between healthy (FSJK) and root rot-affected (FSGF) samples. Red indicates higher abundance, blue indicates lower abundance in diseased samples. Only pathways with significant differences ($q < 0.05$) are shown.

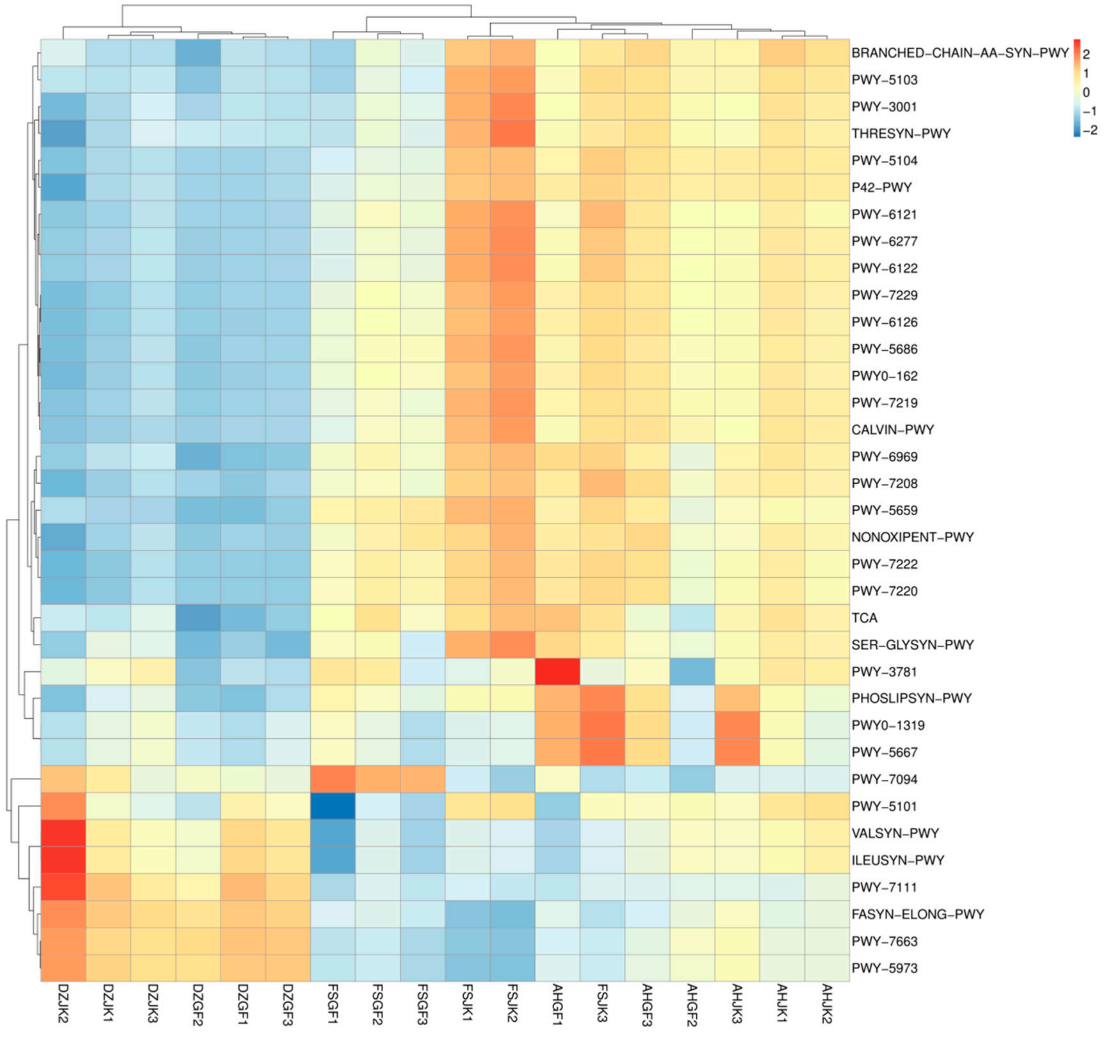

Supplement: Supplementary file 1 [file microorganisms-14-01568-s001.zip › Supplementary Materials.pdf]
